# Quince Gel Attenuates Experimental Ulcerative Colitis Through Antioxidant, Anti-Inflammatory and Mucosal Repair Mechanisms: Integrated Histopathological, Bioinformatic and Molecular Docking Analyses

**DOI:** 10.3390/ph19081161

**Published:** 2026-07-25

**Authors:** Hikmet Özesmer, Eda Yıldızhan

**Affiliations:** 1 Department of General Surgery, Faculty of Medicine, Dicle University, Diyarbakır 21280, Turkey; 2 Department of Histology and Embryology, Faculty of Medicine, Recep Tayyip Erdogan University, Rize 53100, Turkey; eda.yildizhan@erdogan.edu.tr

**Keywords:** ulcerative colitis, quince gel, quercetin, TGF-β, FGF, molecular docking

## Abstract

**Objective:** Ulcerative colitis (UC) is a relapsing inflammatory disorder of the colon in which persistent mucosal injury, oxidative stress, and defective healing processes contribute to disease progression. Given the need for alternative therapeutic strategies, this study evaluated the efficacy of intrarectal quince gel in an experimental UC model using an integrated approach that combined biochemical assays, histopathological and immunohistochemical examinations, bioinformatic analyses, and molecular docking. **Methods:** A total of 28 male Wistar albino rats were randomly allocated to one of four experimental groups: Sham, Quince Gel, UC, and UC + Quince Gel (*n* = 7/group). UC was induced by intrarectal administration of 4% acetic acid, followed by daily intrarectal quince gel treatment for 10 days. To determine the therapeutic effects of quince gel, macroscopic and histopathological changes, colon mass index, oxidative stress indicators (TAS, TOS, OSI, and MDA), serum biochemical parameters, and inflammatory mediators, including TNF-α, IL-1β, and IL-6, were evaluated. TGF-β and FGF expression levels were assessed by immunohistochemistry and quantified using QuPath software (version 0.7.0). The phytochemical composition of quince gel was characterized by LC–MS/MS analysis, while protein–protein interaction network analysis and molecular docking were performed to investigate the potential molecular mechanisms underlying its biological effects. **Results:** Quince gel significantly alleviated acetic acid-induced colonic injury by reducing macroscopic damage scores, histopathological injury, and colon mass index. Treatment restored oxidative balance by increasing total antioxidant status while decreasing total oxidant status, oxidative stress index, and malondialdehyde (MDA) levels. In addition, serum LDH, CRP, TNF-α, IL-1β, and IL-6 levels were markedly reduced compared with the untreated UC group. Histological examination demonstrated preservation of epithelial integrity, reduced inflammatory cell infiltration, and improved mucosal architecture. Quantitative immunohistochemical analysis showed significant attenuation of TGF-β and FGF overexpression following quince gel treatment. LC–MS/MS identified quercetin as a major bioactive constituent of the gel. Bioinformatic analysis revealed TGFB1, STAT3, and MMP9 as central hub proteins within the UC-associated interaction network, whereas molecular docking demonstrated the strongest binding affinity of quercetin toward TNF-α (−7.379 kcal/mol), supporting its potential anti-inflammatory mechanism. **Conclusions:** Quince gel exerts significant anti-inflammatory, antioxidant, and mucosal regenerative effects in experimental UC. The integration of histopathological, immunohistochemical, phytochemical, bioinformatic, and molecular docking findings suggests that quince gel may protect colonic tissue through coordinated modulation of oxidative stress, inflammatory cytokines, and tissue repair pathways. These findings support its potential as a promising complementary therapeutic strategy for UC.

## 1. Introduction

Ulcerative colitis (UC) is a chronic, relapsing inflammatory disorder of the large intestine that primarily affects the colonic mucosa, leading to epithelial damage, mucosal ulceration, and loss of intestinal barrier function [1,2]. Patients with UC typically experience recurrent episodes of abdominal pain, bloody diarrhea, and rectal bleeding, all of which can substantially reduce quality of life and daily functioning [3]. Despite considerable advances in medical management, UC remains a major clinical challenge because of its chronic relapsing course and the limitations of currently available therapies [4].

UC arises from a multifaceted pathogenic process in which genetic predisposition, environmental exposures, gut microbial imbalance, oxidative stress, and aberrant immune regulation interact to promote chronic intestinal inflammation [5,6]. Oxidative stress plays a central role in UC pathogenesis, as excessive generation of reactive oxygen species induces lipid peroxidation and compromises epithelial integrity. Concurrently, elevated levels of pro-inflammatory cytokines, including tumor necrosis factor-α (TNF-α), interleukin (IL)-1β, and IL-6, reinforce the inflammatory response, resulting in persistent mucosal damage and progressive destruction of colonic tissue [5,7,8]. The restoration of mucosal integrity is largely orchestrated by growth factors such as transforming growth factor-β (TGF-β) and fibroblast growth factor (FGF). These mediators promote epithelial regeneration, coordinate extracellular matrix remodeling, and facilitate recovery of the intestinal barrier following injury [9,10]. However, persistent activation of these pathways during chronic inflammation may contribute to abnormal tissue remodeling and fibrosis [11,12,13]. Current therapeutic options for UC, such as aminosalicylates, corticosteroids, immunomodulators, and biologic therapies, have significantly enhanced disease management. Nevertheless, their long-term effectiveness is frequently compromised by adverse reactions, inconsistent clinical responses, and high treatment costs [14]. As a result, considerable attention has shifted toward naturally occurring compounds with anti-inflammatory and antioxidant properties that could provide safe and effective complementary strategies for the management of UC [15].

Traditionally, *Cydonia oblonga* (quince) has been employed as a medicinal plant for gastrointestinal ailments and wound care [16]. Phytochemical investigations have identified quince as a rich source of flavonoids and phenolic constituents that exhibit pronounced antioxidant and anti-inflammatory activities. Moreover, experimental studies have demonstrated that quince gel formulations promote tissue regeneration and accelerate healing of damaged tissues [17,18,19]. Previous experimental studies have demonstrated that *Cydonia oblonga* possesses gastroprotective, antioxidant, and anti-inflammatory properties. Quince extracts have been reported to attenuate gastric and intestinal injury, reduce oxidative stress, and suppress inflammatory responses in experimental gastrointestinal disease models [20,21]. These biological effects have been largely attributed to its phenolic constituents, including quercetin, chlorogenic acid, caffeic acid, rutin, and kaempferol, which have individually been shown to modulate oxidative stress, inflammatory cytokine production, and intestinal barrier function in experimental colitis and related inflammatory disorders [22,23,24,25]. Collectively, these findings provide a biological rationale for investigating quince gel as a potential therapeutic approach for experimental ulcerative colitis. Nevertheless, the effects of quince gel on intestinal mucosal healing and the molecular mechanisms underlying its potential therapeutic actions in UC have not been comprehensively investigated.

To investigate the therapeutic potential of quince gel, we employed an experimental UC model and performed a comprehensive evaluation combining biochemical, histopathological, immunohistochemical, phytochemical, bioinformatic, and molecular docking analyses. In addition to assessing oxidative stress and inflammatory responses, this study investigated TGF-β- and FGF-mediated mucosal repair pathways, identified the major bioactive constituent of quince gel by LC–MS/MS analysis, and explored its potential molecular mechanisms through protein–protein interaction network analysis and molecular docking. This integrated approach provides novel mechanistic insight into the therapeutic potential of quince gel as a complementary treatment strategy for UC.

## 2. Results

### 2.1. LC–MS/MS Analysis of Quince Gel

LC–MS/MS analysis identified several polyphenolic compounds in the quince gel, including caffeic acid, chlorogenic acid, rutin, quercetin, and kaempferol (Table 1; Supplementary Figure S1). Quercetin was detected at a retention time of 8.74 min with a molecular ion of *m*/*z* 301.03, while chlorogenic acid, caffeic acid, rutin, and kaempferol exhibited retention times of 5.62, 4.81, 7.95, and 9.26 min and molecular ions of *m*/*z* 353.08, 179.03, 609.14, and 285.04, respectively. The total ion chromatogram (TIC) demonstrated that quercetin exhibited the highest relative peak intensity among the detected compounds, whereas rutin and kaempferol were also present as prominent constituents (Supplementary Figure S1). Based on its dominant chromatographic signal together with its well-established antioxidant and anti-inflammatory properties, quercetin was selected as the representative phytochemical for subsequent molecular docking analysis. These LC–MS/MS findings provide phytochemical support for the biological effects of quince gel and establish a rationale for the subsequent in silico analyses.

### 2.2. Effects of Quince Gel on Oxidative Stress Parameters

The oxidative stress parameters are summarized in Table 2. Kruskal–Wallis analysis demonstrated significant differences among the four groups for TAS, TOS, OSI, and MDA levels (all *p* < 0.001). The UC group exhibited the lowest TAS levels and the highest TOS, OSI, and MDA values, indicating marked oxidative stress compared with the Sham and Quince Gel groups. Administration of quince gel in rats with UC significantly ameliorated oxidative imbalance, as evidenced by increased TAS levels and decreased TOS, OSI, and MDA values compared with the untreated UC group. The Quince Gel group did not differ significantly from the Sham group, suggesting that quince gel administration alone did not adversely affect systemic oxidative status.

### 2.3. Effects of Quince Gel on Biochemical and Inflammatory Parameters

Serum biochemical and inflammatory markers also differed significantly among the experimental groups (Table 3). Calcium, albumin, CRP, LDH, TNF-α, IL-1β, and IL-6 levels showed significant intergroup differences (all *p* ≤ 0.002). The UC group demonstrated significantly elevated calcium, CRP, LDH, TNF-α, IL-1β, and IL-6 levels, accompanied by decreased albumin concentrations, reflecting severe systemic inflammation and tissue injury. In contrast, treatment with quince gel significantly attenuated these alterations in the UC + Quince Gel group. Although most inflammatory biomarkers remained higher than those observed in the Sham group, their levels were markedly reduced compared with those in untreated UC animals. The Quince Gel group showed biochemical and inflammatory profiles comparable to those of the Sham group.

### 2.4. Effects of Quince Gel on Macroscopic and Histopathological Findings

Macroscopic damage score, colon mass index, and histopathological score were significantly different among the groups (all *p* ≤ 0.001) (Table 4). The UC group exhibited the highest macroscopic damage and histopathological scores together with an increased colon mass index, indicating severe colonic injury and inflammation. Quince gel treatment significantly reduced macroscopic damage and histopathological injury severity and partially normalized the colon mass index compared with the untreated UC group. Animals receiving quince gel alone showed no evidence of significant colonic injury and demonstrated findings comparable to those of the Sham group.

### 2.5. Histopathological Findings

**Figure 1:**
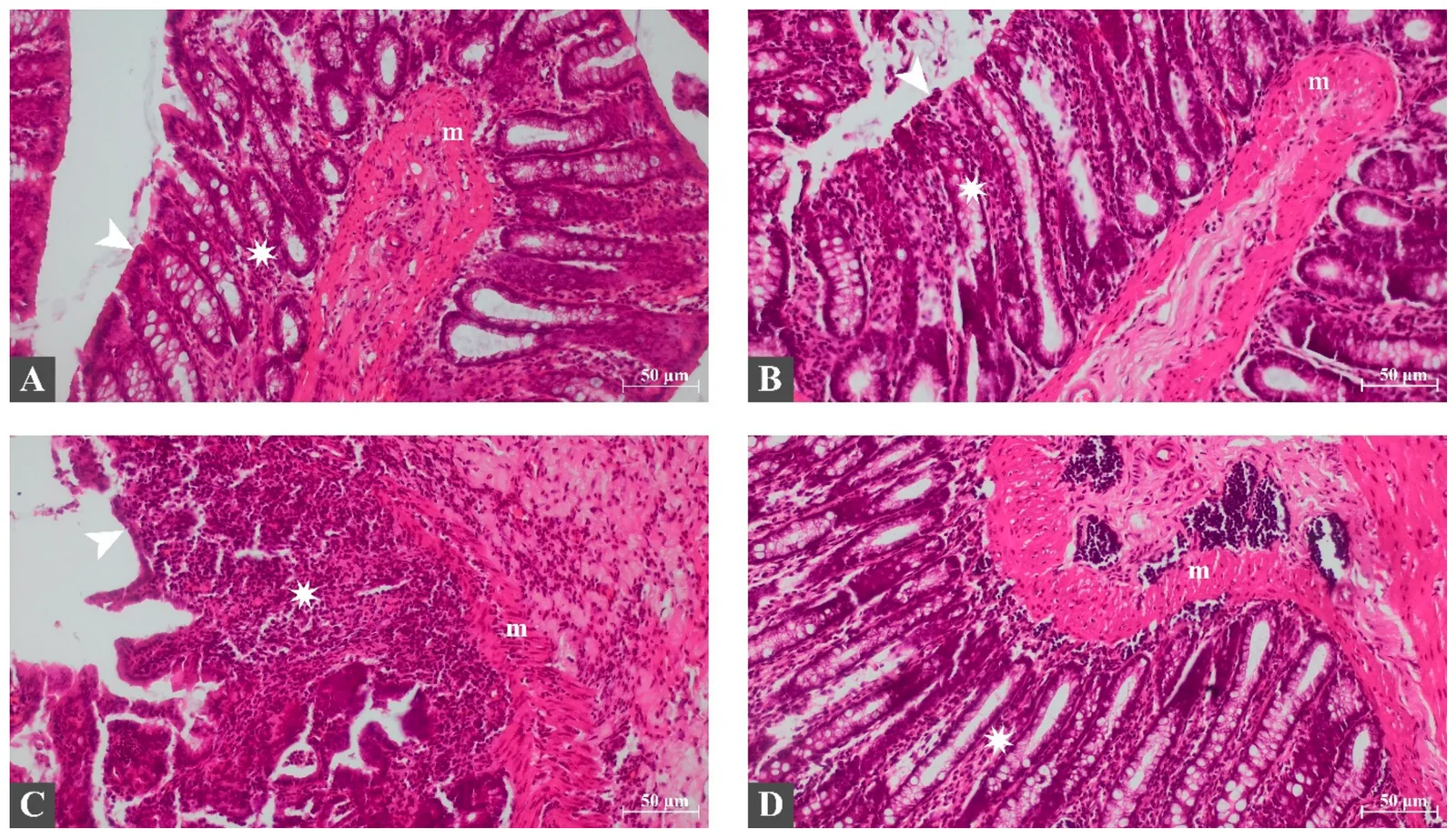
Histopathological evaluation of colonic tissue by hematoxylin and eosin staining. Representative micrographs of the experimental groups. (**A**) Sham group, (**B**) Quince Gel group, (**C**) UC, (**D**) UC + Quince Gel. Arrowhead: lamina epithelialis; asterisk: lamina propria; m: muscularis mucosae. Hematoxylin and eosin staining; scale bar = 50 μm.

Representative hematoxylin and eosin-stained colon sections are shown in Figure 1. The sham and quince gel groups exhibited preserved colonic architecture with intact surface epithelium, regularly arranged crypts, and minimal inflammatory cell infiltration within the lamina propria. In contrast, the UC group demonstrated marked histopathological alterations characterized by disruption of the mucosal epithelium, distortion of crypt architecture, and dense inflammatory cell infiltration expanding the lamina propria. Treatment with quince gel markedly attenuated these pathological changes, as evidenced by improved mucosal organization, more regular crypt morphology, and reduced inflammatory cell infiltration compared with the untreated UC group, although complete histological recovery was not observed. These microscopic findings were consistent with the significantly lower histopathological scores observed in the UC + quince gel group compared with the UC group (Table 4).

### 2.6. Quince Gel Attenuates Increased TGF-β Immunoreactivity in Experimental Ulcerative Colitis

**Figure 2:**
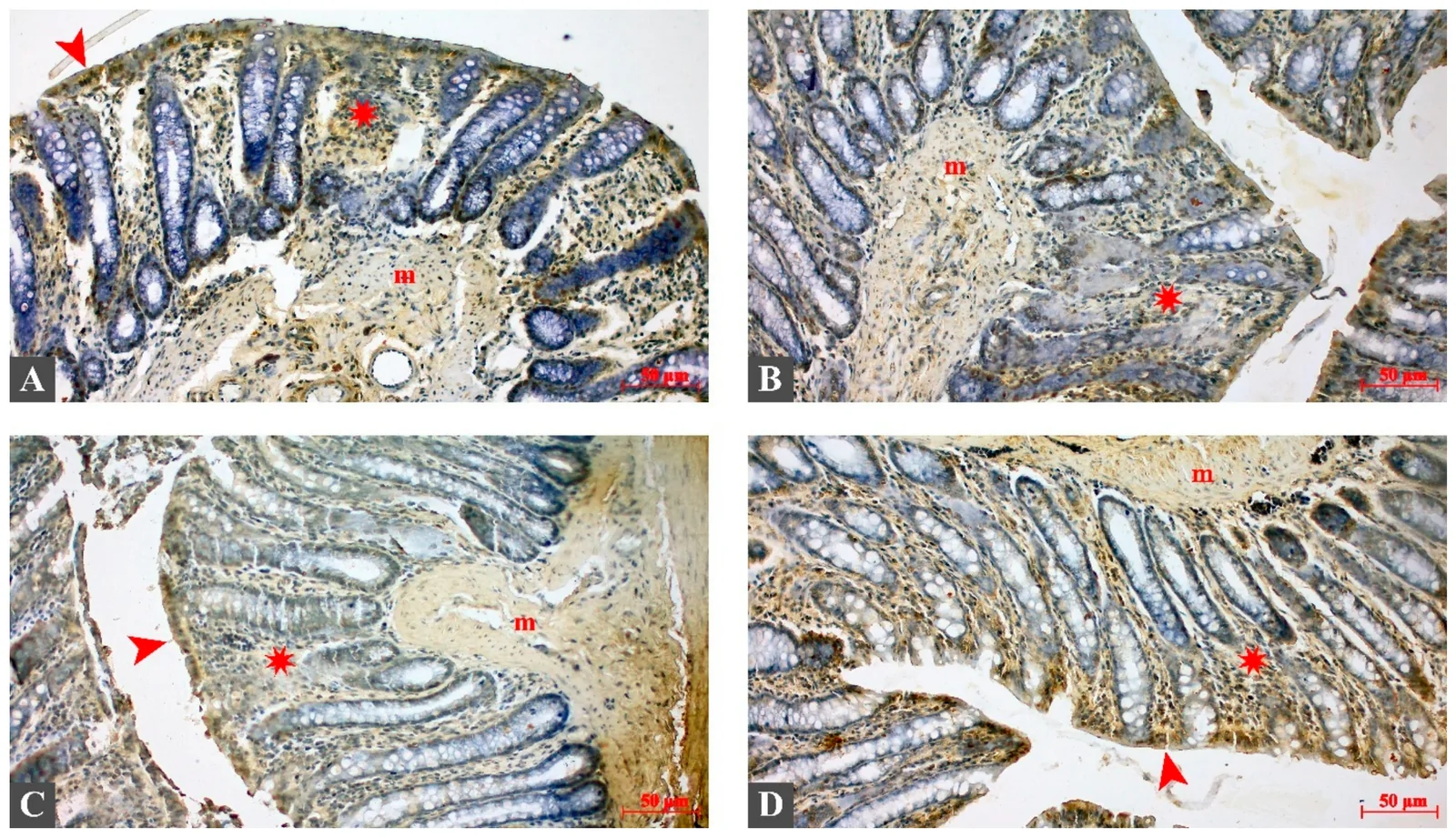
Immunohistochemical evaluation of TGF-β expression in colonic tissues. Representative micrographs of the experimental groups. (**A**) Sham group, (**B**) Quince Gel group, (**C**) UC group, (**D**) UC + Quince Gel group. Arrowhead: lamina epithelialis; asterisk: lamina propria; m: muscularis mucosae. Immunohistochemical staining for TGF-β; scale bar = 50 μm.

Immunohistochemical evaluation of TGF-β expression revealed distinct differences among the experimental groups (Figure 2). In the sham group, weak TGF-β immunoreactivity was observed predominantly in the surface epithelium and lamina propria. A similar low-intensity staining pattern was detected in the quince gel group, indicating that gel administration alone did not alter basal TGF-β expression. In contrast, the UC group exhibited increased TGF-β immunoreactivity, particularly within the lamina propria and areas associated with inflammatory cell infiltration and mucosal injury. In the UC + quince gel group, TGF-β staining intensity was reduced compared with the UC group and displayed a more regular distribution, accompanied by preservation of the mucosal architecture and decreased inflammatory infiltration. These findings suggest that topical quince gel treatment attenuated the inflammation-associated increase in TGF-β expression while supporting mucosal tissue repair (Figure 2).

**Figure 3:**
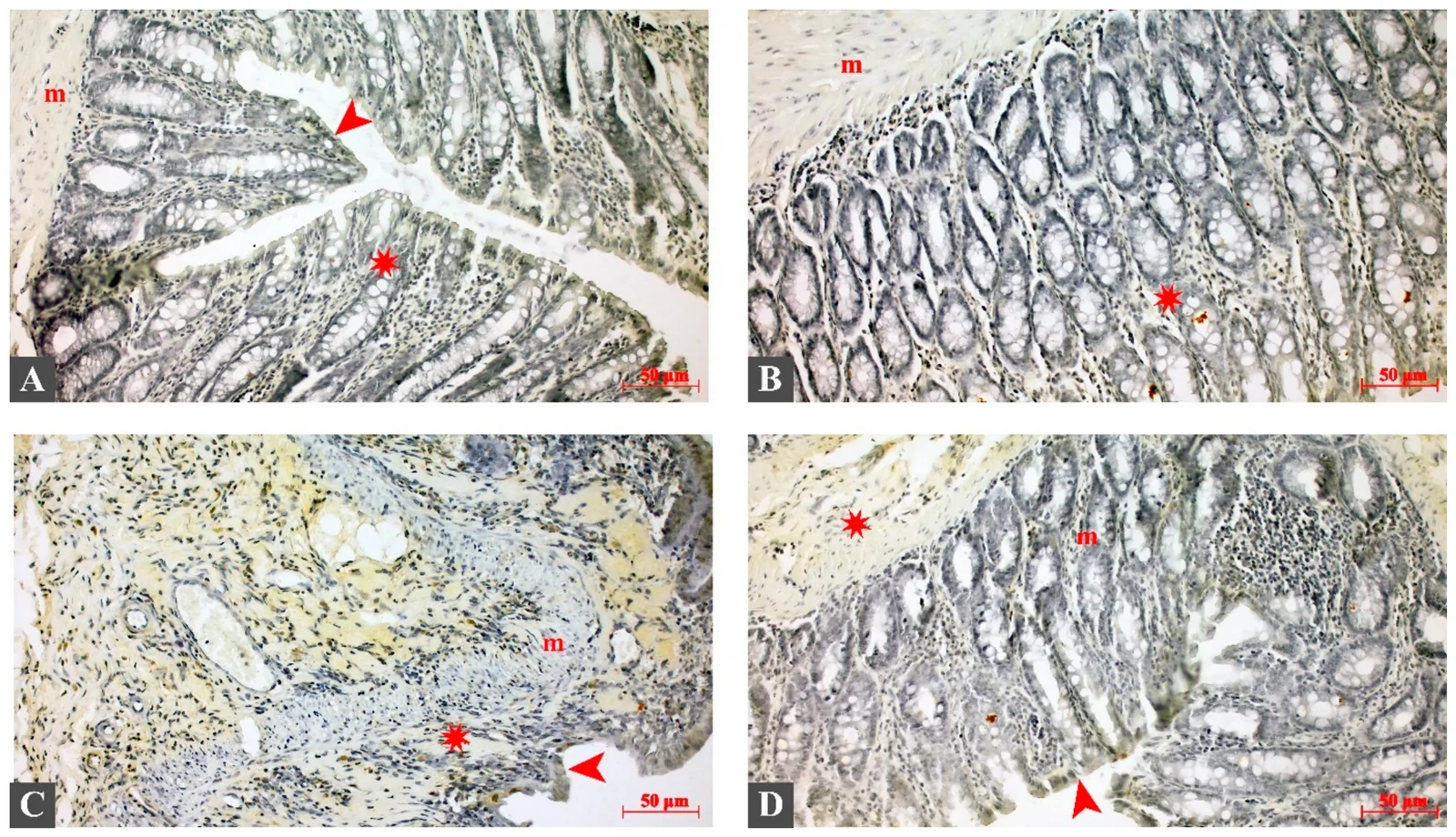
Representative immunohistochemical staining for FGF in the experimental groups. (**A**) Sham group, (**B**) Quince Gel group, (**C**) UC group, (**D**) UC + Quince Gel group. Arrowhead: lamina epithelialis; asterisk: lamina propria; m: muscularis mucosae. Immunohistochemical staining for FGF; scale bar = 50 μm.

Immunohistochemical analysis demonstrated differences in FGF expression among the experimental groups (Figure 3). In the Sham group, weak FGF immunoreactivity was observed predominantly in the epithelial lining and crypt structures. The Quince Gel group exhibited a staining pattern comparable to the Sham group, with low basal FGF expression and preserved tissue architecture. In the UC group, FGF immunoreactivity was increased, particularly in the damaged mucosal epithelium, crypt regions, and areas associated with inflammatory cell infiltration, consistent with an activated tissue repair response. In the UC + Quince Gel group, FGF expression was reduced compared with the UC group and showed a more regular distribution within the mucosa, although immunoreactivity remained slightly higher than that observed in the Sham group. These findings indicate that Quince Gel treatment attenuated the injury-associated increase in FGF expression while supporting restoration of mucosal integrity (Figure 3).

### 2.7. Quantitative Analysis of TGF-β and FGF Expression

Quantitative immunohistochemical analysis revealed significant differences in both TGF-β and FGF expression among the experimental groups (Table 5). The UC group exhibited the highest TGF-β-positive area (36.7 ± 5.4%) and H-score (175.8 ± 18.9), compared with the Sham group (7.4 ± 1.8% and 48.3 ± 9.5, respectively). Similarly, FGF expression was markedly increased in the UC group, with a positive staining area of 33.5 ± 4.9% and an H-score of 168.2 ± 17.4, whereas the corresponding values in the Sham group were 6.9 ± 1.5% and 44.2 ± 8.7.

Treatment with quince gel significantly reduced the immunoreactivity of both markers following UC induction. In the UC + Quince Gel group, the TGF-β-positive area and H-score decreased to 20.5 ± 3.6% and 101.4 ± 14.7, respectively, while the FGF-positive area and H-score were reduced to 18.7 ± 3.2% and 96.3 ± 13.2. Despite these reductions, expression levels remained moderately higher than those observed in the Sham group.

No appreciable differences were observed between the Sham and Quince Gel groups, which exhibited comparable TGF-β and FGF staining patterns. Overall, the quantitative immunohistochemical findings were consistent with the qualitative microscopic observations and demonstrated attenuation of the UC-associated increase in TGF-β and FGF expression following quince gel treatment.

### 2.8. Highly Interconnected Protein–Protein Interaction Network Among Inflammation- and Tissue Repair-Related Targets

**Figure 4:**
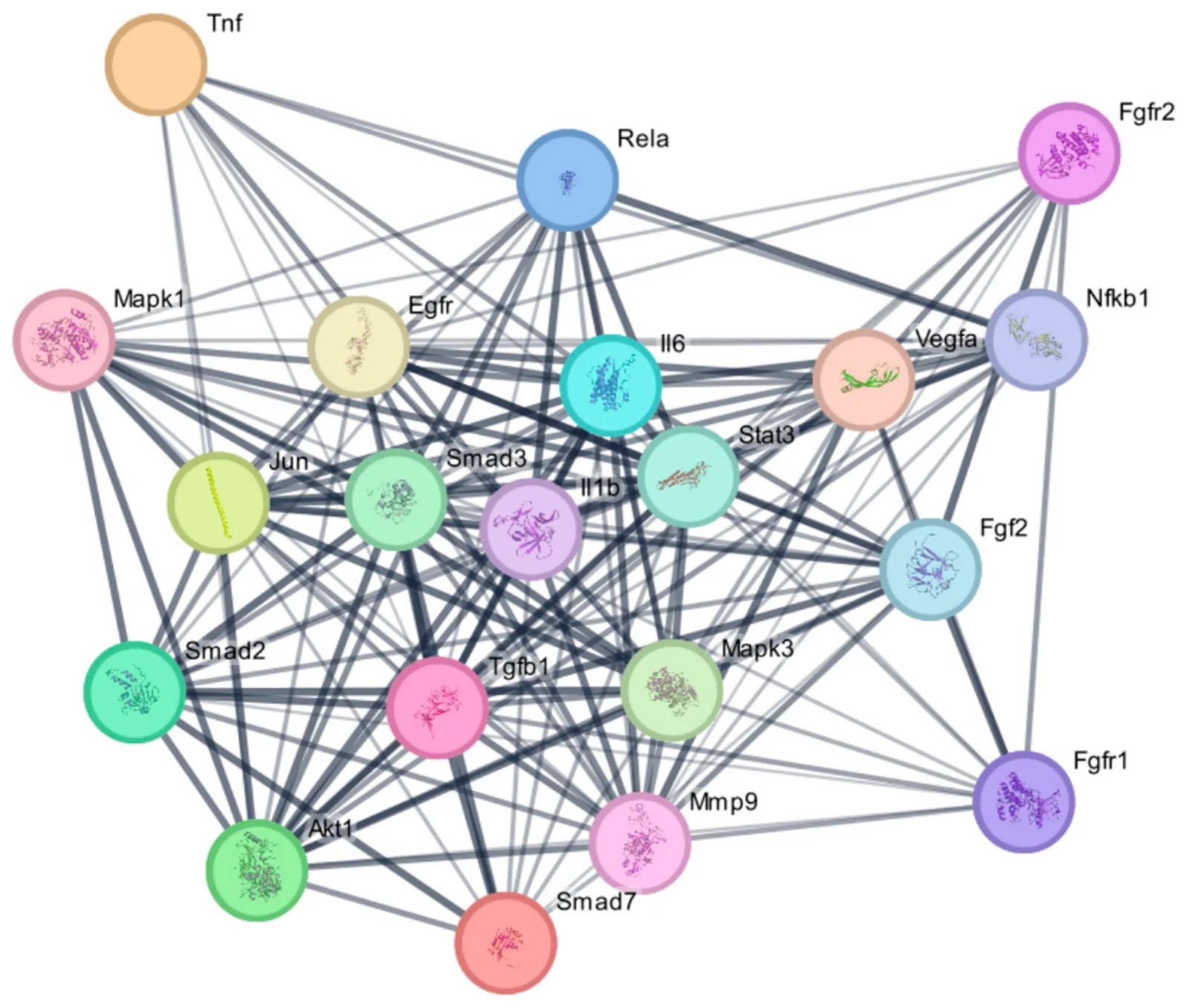
Protein–protein interaction network of inflammation- and tissue repair-related targets associated with UC. The network was generated using the STRING database and visualized in Cytoscape. Nodes represent proteins, and edges indicate predicted functional associations. The dense connectivity among TGFB1, STAT3, IL6, IL1B, RELA, AKT1, and SMAD family members highlights the coordinated regulation of inflammatory signaling and mucosal repair pathways.

Protein–protein interaction (PPI) network analysis of inflammation- and tissue repair-related targets generated a highly interconnected network comprising 20 nodes and 151 edges, with an average node degree of 15.1, network density of 0.795, clustering coefficient of 0.864, and characteristic path length of 1.205, indicating extensive functional interactions among the analyzed proteins (Figure 4).

Degree centrality analysis identified TGFB1, MMP9, and STAT3 as the principal hub genes, each with a degree value of 19, followed by IL6, MAPK3, and AKT1 (degree = 18) and IL1B (degree = 17). Additional highly connected nodes included JUN, EGFR, and FGF2, each exhibiting a degree value of 16 (Table 6).

### 2.9. Molecular Docking Results

Molecular docking analysis was performed to evaluate the potential interactions between quercetin and selected inflammation- and tissue remodeling-related target proteins (Table 7). Among the analyzed targets, quercetin exhibited the strongest predicted binding affinity toward TNF-α (−7.379 kcal/mol), followed by TGF-β1 (−7.059 kcal/mol), IL-1β (−6.913 kcal/mol), IL-6 (−6.434 kcal/mol), and FGF2 (−5.682 kcal/mol). Based on these results, the quercetin–TNF-α complex was selected for subsequent structural interaction analysis and molecular visualization.

**Figure 5:**
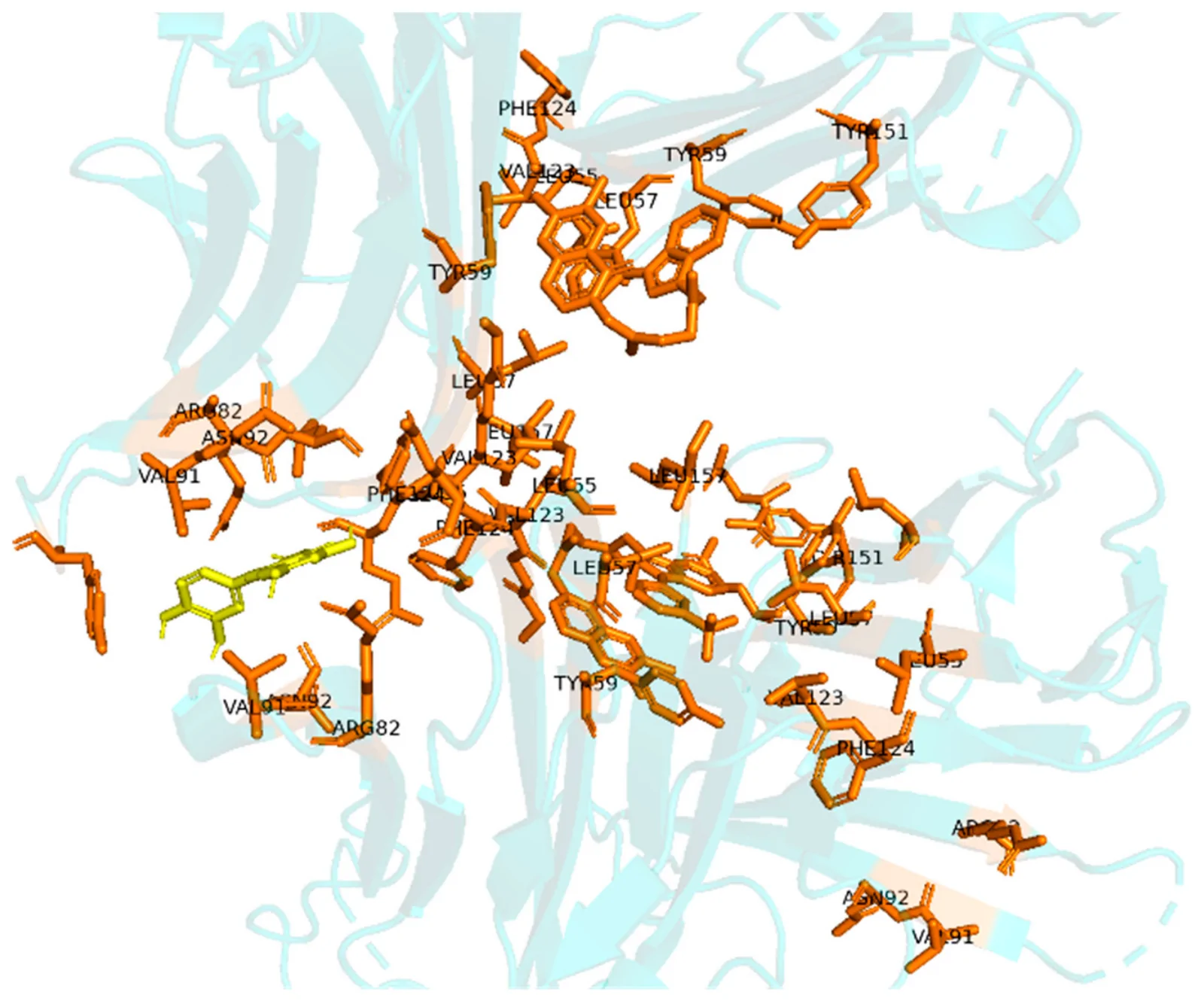
Three-dimensional molecular docking model illustrating the interaction of quercetin with the TNF-α binding pocket. Quercetin is shown as stick representation (yellow), while the surrounding amino acid residues involved in the predicted binding interface are highlighted as orange sticks, and the TNF-α structure is displayed in cartoon representation. The best-ranked docking pose exhibited a binding affinity of −7.379 kcal/mol.

Three-dimensional molecular docking analysis predicted that quercetin occupies a surface-accessible binding pocket of TNF-α (Figure 5). The ligand was positioned in proximity to several amino acid residues, including Leu55, Leu57, Tyr59, Arg82, Val91, Asn92, Val123, Phe124, Gln125, Tyr151, Ile155, and Leu157, suggesting the potential formation of multiple non-covalent interactions that could contribute to stabilization of the ligand–protein complex. The predicted binding pose of quercetin within the binding cavity was consistent with its calculated binding affinity and provides computational support for a potential interaction with TNF-α.

## 3. Discussion

The present study demonstrated that intrarectal administration of quince gel significantly attenuated experimental UC by reducing oxidative stress, suppressing inflammatory responses, and improving mucosal architecture. In addition to ameliorating macroscopic and histopathological injury, quince gel decreased the expression of pro-inflammatory cytokines and modulated the expression of TGF-β and FGF, which are closely associated with tissue repair. Furthermore, LC–MS/MS analysis identified quercetin as a major bioactive constituent of the gel, while bioinformatic network analysis and molecular docking provided complementary computational evidence supporting the experimental findings and suggesting potential molecular mechanisms. Collectively, these results suggest that quince gel exerts its protective effects through coordinated regulation of oxidative stress, inflammation, and mucosal healing pathways.

Oxidative stress is recognized as one of the principal mechanisms contributing to the pathogenesis and progression of UC through excessive production of reactive oxygen species and subsequent disruption of intestinal epithelial integrity [26,27]. In the present study, experimental UC markedly decreased total antioxidant status while significantly increasing total oxidant status, oxidative stress index, and MDA levels, indicating severe oxidative injury. Treatment with quince gel effectively restored the oxidant–antioxidant balance by increasing antioxidant capacity and reducing lipid peroxidation, suggesting a substantial attenuation of oxidative stress.

These findings are consistent with previous studies demonstrating that oxidative stress is closely associated with disease severity in UC and that antioxidant therapies may alleviate mucosal injury by limiting reactive oxygen species-mediated cellular damage [28,29,30]. Sahoo et al. [31] reported that excessive oxidative stress contributes to intestinal inflammation and epithelial barrier dysfunction, whereas enhancement of endogenous antioxidant defense mechanisms may improve mucosal healing. Similarly, Gubitosa et al. [32] demonstrated that *Cydonia oblonga*-derived bioactive compounds exert significant antioxidant and anti-inflammatory effects by reducing oxidative damage and inflammatory signaling in injured cells.

The antioxidant activity observed in the present study may be attributed to the rich polyphenolic composition of quince gel. Our LC–MS/MS analysis identified quercetin together with chlorogenic acid, caffeic acid, rutin, and kaempferol as major phytochemical constituents, all of which have well-documented free radical scavenging properties [33,34]. The improvement in TAS, TOS, OSI, and MDA levels therefore provides biochemical evidence that quince gel attenuates oxidative stress, which may represent one of the primary mechanisms underlying its protective effects against experimental UC [20,22].

Inflammatory cytokines play a central role in the initiation and progression of UC by promoting epithelial injury, leukocyte recruitment, and amplification of the mucosal inflammatory response [35]. In the present study, experimental UC resulted in marked increases in TNF-α, IL-1β, and IL-6 levels, whereas treatment with quince gel significantly reduced the expression of these pro-inflammatory mediators. These findings indicate that quince gel effectively attenuates the inflammatory response associated with colonic injury [19].

Our results are in agreement with previous studies demonstrating that excessive production of TNF-α, IL-1β, and IL-6 is closely associated with disease activity and tissue damage in UC [36,37]. Strober and Fuss [38] emphasized the pivotal role of these cytokines in sustaining chronic intestinal inflammation, while recent evidence has shown that therapeutic suppression of cytokine signaling represents one of the most effective strategies for controlling disease progression. Therefore, the reduction in these inflammatory mediators observed in the present study may represent an important mechanism contributing to the protective effects of quince gel.

Interestingly, the molecular docking analysis provided additional mechanistic support for these experimental findings. Among the evaluated target proteins, quercetin—the major flavonoid identified by LC–MS/MS analysis [39]—exhibited the strongest predicted binding affinity toward TNF-α, surpassing its interactions with TGF-β1, IL-1β, IL-6, and FGF2. This favorable binding profile suggests that quercetin may interact with TNF-α, although the biological relevance of this predicted interaction requires experimental validation. Although these computational predictions require further experimental validation, they are consistent with the significant reduction in tissue TNF-α levels observed following quince gel treatment and provide complementary computational support for the anti-inflammatory potential of its phytochemical constituents.

Histopathological evaluation further confirmed the protective effects of quince gel on colonic tissue integrity. Experimental UC was characterized by extensive epithelial disruption, crypt distortion, inflammatory cell infiltration, and increased macroscopic and microscopic injury scores, whereas quince gel treatment markedly improved mucosal architecture and reduced tissue damage. These observations were accompanied by a significant reduction in the colon mass index, indicating attenuation of inflammatory edema and tissue swelling.

The beneficial histological effects of quince gel were paralleled by changes in the expression of the tissue repair-associated growth factors TGF-β and FGF [40]. Quantitative immunohistochemical analysis demonstrated marked upregulation of both markers in the UC group, whereas quince gel treatment significantly reduced their positive staining areas and H-scores. Although the expression levels remained moderately higher than those observed in the sham group, they were substantially lower than those in untreated UC animals, suggesting partial normalization of the tissue repair response.

TGF-β and FGF are essential regulators of epithelial regeneration, extracellular matrix remodeling, and wound healing within the intestinal mucosa [41]. Previous studies have shown that transient activation of these pathways contributes to mucosal repair following injury, whereas persistent overexpression may promote pathological remodeling and fibrosis. Beck et al. [42] demonstrated that TGF-β signaling plays a critical role in intestinal epithelial restitution, while Rodari et al. [43] emphasized that dysregulated TGF-β activity may contribute to chronic inflammatory disorders through aberrant tissue remodeling. Likewise, members of the FGF family are known to stimulate epithelial proliferation and angiogenesis during mucosal healing [44].

The reduction in TGF-β and FGF expression observed after quince gel treatment should therefore be interpreted as reflecting attenuation of excessive injury-associated activation rather than inhibition of physiological repair mechanisms. This interpretation is supported by the concomitant improvement in histopathological findings, preservation of epithelial architecture, and reduction in inflammatory infiltration. Collectively, these findings indicate that quince gel not only suppresses inflammation but also contributes to restoration of mucosal homeostasis through balanced regulation of tissue repair pathways. Therefore, the observed modulation of TGF-β and FGF expression is likely to reflect restoration of tissue homeostasis rather than complete suppression of regenerative signaling, which remains essential for normal mucosal healing.

The integration of phytochemical characterization, bioinformatic network analysis, and molecular docking represents one of the major strengths of the present study and provides complementary mechanistic support for the experimental findings. LC–MS/MS analysis identified quercetin as one of the principal bioactive flavonoids present in quince gel, together with chlorogenic acid, caffeic acid, rutin, and kaempferol [23]. These phytochemicals have been widely reported to possess antioxidant and anti-inflammatory properties and may collectively contribute to the biological activity of the gel rather than acting through a single constituent.

To further explore the molecular basis of these protective effects, protein–protein interaction network analysis was performed using inflammation- and tissue repair-related targets associated with UC. The network analysis identified TGFB1, STAT3, MMP9, IL6, and AKT1 as the major hub proteins, highlighting their central roles within the interconnected signaling pathways regulating inflammation and mucosal repair. These findings are consistent with the experimental observations demonstrating altered expression of inflammatory cytokines and growth factor-related markers in colonic tissue [45], suggesting that the protective effects of quince gel may involve modulation of multiple molecular pathways simultaneously.

Molecular docking analysis provided additional support for this hypothesis by showing that quercetin exhibited the strongest predicted binding affinity toward TNF-α among the analyzed targets. Considering the pivotal role of TNF-α in initiating and sustaining intestinal inflammation [46], this predicted interaction is consistent with the significant reduction in tissue TNF-α levels observed following quince gel treatment, although a direct causal relationship cannot be inferred from the present data. Nevertheless, it is likely that the biological activity of quince gel results from the combined actions of multiple phytochemicals acting on interconnected signaling pathways rather than from the effect of quercetin alone. The convergence of the phytochemical, bioinformatic, molecular docking, and experimental findings therefore strengthens the hypothesis that quince gel exerts its protective effects through coordinated regulation of oxidative stress, inflammatory signaling, and mucosal repair mechanisms.

Several limitations of the present study should be acknowledged. First, only a single dose of quince gel was evaluated, and no positive control group treated with a standard anti-colitic agent, such as mesalazine, was included. Therefore, neither the optimal therapeutic dose nor the relative efficacy of quince gel compared with established therapies could be determined. Second, the LC–MS/MS analysis was designed for phytochemical characterization and relative abundance assessment rather than absolute quantification. Consequently, the individual contributions and potential synergistic interactions of the identified phenolic constituents remain to be elucidated through dedicated quantitative phytochemical studies. Third, the protein–protein interaction network analysis and molecular docking investigations provide complementary in silico evidence and require further experimental validation using molecular techniques such as Western blotting, RT-qPCR, or ligand–protein binding assays. Finally, long-term biosafety, formulation optimization, pharmacokinetic characteristics, and colonic retention of intrarectal quince gel were not evaluated in this proof-of-concept study. Future investigations should address these aspects, together with intestinal barrier integrity, gut microbiota composition, and comparative efficacy against standard therapies, to further clarify the mechanisms of action and support the potential clinical translation of quince gel.

Although the present study demonstrated significant anti-inflammatory and mucosal protective effects of quince gel, the findings should be interpreted as proof-of-concept evidence rather than direct evidence of superiority or equivalence to currently available therapies. Comparative studies including established anti-colitic agents such as mesalazine or sulfasalazine are warranted to determine the relative therapeutic efficacy of quince gel and its potential role as an adjunctive or complementary treatment.

## 4. Materials and Methods

### 4.1. Ethical Approval

Ethical approval for all experimental procedures was obtained from the Dicle University Animal Ethics Committee (Protocol No. 2025/02; 26 March 2025; Türkiye). Throughout the study, animal handling and experimental procedures complied with the Guide for the Care and Use of Laboratory Animals as well as internationally accepted standards for animal welfare and ethical research [47].

### 4.2. Plant Material and Gel Preparation

Fresh ripe quince (*Cydonia oblonga* Mill.) fruits were purchased from a local market in Diyarbakır, Türkiye. The fruits were washed thoroughly with distilled water, and the seeds were manually separated under aseptic conditions. Quince gel was prepared by soaking 10 seeds in 10 mL of sterile distilled water overnight (approximately 16 h) at room temperature to allow natural mucilage formation. The hydrated mucilage was carefully separated from the seeds and used directly without further chemical processing or additives. Fresh gel was prepared daily throughout the experimental period and stored at 4 °C until use [19].

### 4.3. Phytochemical Characterization of Quince Gel by LC–MS/MS

Prior to the in vivo experiments, the phytochemical profile of the quince gel was investigated using liquid chromatography–tandem mass spectrometry (LC–MS/MS). Analyses were carried out on an Agilent 6460 Triple Quadrupole LC–MS/MS system (Agilent Technologies, Santa Clara, CA, USA) equipped with an electrospray ionization (ESI) source operated in the negative ionization mode. Separation of the analytes was achieved on a reverse-phase C18 column (150 × 4.6 mm, 5 μm) maintained at 30 °C. The chromatographic system employed a binary mobile phase consisting of 0.1% formic acid in water (solvent A) and 0.1% formic acid in acetonitrile (solvent B). Elution was performed using a gradient program at a constant flow rate of 0.30 mL/min, and 5 μL of each sample was injected into the system.

Phytochemical identification was established by integrating retention times, precursor ion masses (*m*/*z*), characteristic fragmentation patterns, and comparisons with authentic reference standards as well as published mass spectral databases. Because flavonoids are recognized for their antioxidant and anti-inflammatory properties, particular attention was directed toward this class of compounds. LC–MS/MS analysis identified quercetin as one of the predominant bioactive constituents of the quince gel, and this compound was subsequently selected for molecular docking analysis [48,49].

### 4.4. Experimental Animals and Study Design

This study included 28 adult male Wistar albino rats aged 8–10 weeks. Animals were maintained under controlled laboratory conditions, including a temperature of 22 ± 3 °C and a 12 h light/12 h dark cycle, with unrestricted access to standard pellet diet and water during the entire experimental period. They were then randomly allocated into four groups, with seven animals in each group:

- Sham group: Animals received 1 mL of physiological saline intrarectally once daily for 10 consecutive days.
- Quince Gel group: Animals were treated with 1 mL of quince gel administered intrarectally once daily for 10 consecutive days.
- UC group: Experimental ulcerative colitis was established by intrarectal instillation of 1 mL of 4% acetic acid. No further treatment was administered following colitis induction.
- UC + Quince Gel group: Ulcerative colitis was induced using 1 mL of 4% acetic acid administered intrarectally. Twenty-four hours after induction, rats received 1 mL of quince gel intrarectally once daily for 10 consecutive days.

### 4.5. Induction of Experimental UC and Quince Gel Treatment

Experimental ulcerative colitis was established using the acetic acid model as previously reported [50,51]. Prior to the procedure, rats were fasted overnight and anesthetized. Colitis was induced by intrarectal instillation of 1 mL of 4% acetic acid (AA; pH 2.4) through a pediatric catheter advanced approximately 8 cm from the anal verge. To ensure homogeneous distribution of the acetic acid throughout the distal colon, approximately 1 mL of air was subsequently injected, and the animals were maintained in the Trendelenburg position for 1 min to minimize leakage [50]. Development of colitis was verified 24 h after AA administration based on the presence of rectal bleeding and diarrhea. This experimental model reproduces many of the histopathological and inflammatory characteristics observed in human UC [52,53].

Quince gel was prepared following the protocol described by Ermiş et al. [19]. Treatment was initiated 24 h after colitis induction, and rats assigned to the treatment groups received 1 mL of quince gel intrarectally once daily for 10 consecutive days. The gel was administered using a pediatric catheter inserted approximately 8 cm into the distal colon.

### 4.6. Sample Collection, Macroscopic Evaluation, and Colon Mass Index

At the completion of the 10-day treatment protocol, food was withheld overnight before all animals were anesthetized with ketamine hydrochloride (90 mg/kg; Ketalar, Pfizer Inc., New York, NY USA) and xylazine hydrochloride (10 mg/kg; Rompun, Bayer Health Care AG, Leverkusen, Germany). Euthanasia was subsequently performed by exsanguination under deep anesthesia in accordance with national and international recommendations for the ethical use of laboratory animals. Blood samples were obtained by cardiac puncture into biochemical collection tubes and allowed to clot at room temperature for 10 min. The samples were then centrifuged at 3000 rpm for 15 min using a refrigerated centrifuge (Rotanta 460, Hettich, Tuttlingen, Germany), and the resulting serum was separated and stored at −80 °C until biochemical analyses were performed. Following euthanasia, the entire colon was carefully excised, rinsed with physiological saline to remove luminal contents, gently dried with absorbent paper, and weighed for calculation of the colon mass index as described by Soliman et al. [54]. The distal 8 cm portion of the colon was subsequently opened along its longitudinal axis and irrigated with 0.9% saline to eliminate residual fecal material and blood.

Gross colonic injury was independently assessed by blinded observers using a previously published macroscopic scoring system [55]: 0, normal appearance; 1, mucosal erythema; 2, mild edema with bleeding or superficial erosion; 3, moderate edema associated with ulceration or erosion; and 4, severe edema accompanied by extensive ulceration, erosion, and necrosis. Following macroscopic assessment, distal colon specimens were fixed in 10% neutral-buffered formalin for subsequent histopathological and immunohistochemical examinations.

### 4.7. Biochemical Analyses

At the end of the experimental protocol, blood samples were collected and centrifuged to separate serum. The serum was aliquoted and preserved at −80 °C until biochemical analyses were performed.

Serum levels of C-reactive protein (CRP; Cat. No. E0053Ra, BT Laboratory, Jiaxing, China), malondialdehyde (MDA; Cat. No. E0156Ra, BT Laboratory, Jiaxing, China), albumin (Cat. No. E1276Ra, BT Laboratory, Jiaxing, China), tumor necrosis factor-α (TNF-α; Cat. No. E0764Ra, BT Laboratory, Jiaxing, China), interleukin-1β (IL-1β; Cat. No. E0119Ra, BT Laboratory, Jiaxing, China), interleukin-6 (IL-6; Cat. No. E0135Ra, BT Laboratory, Jiaxing, China), lactate dehydrogenase (LDH; Cat. No. E-EL-R2547, Elabscience, Houston, TX, USA), and calcium (Cat. No. E-BC-K103-M, Elabscience, Houston, TX, USA) were quantified using commercially available rat-specific ELISA kits in accordance with the manufacturers’ protocols.

Before analysis, all serum samples were equilibrated to room temperature and analyzed in duplicate. Standards and samples were dispensed into antibody-coated microplates, followed by sequential incubation, washing, and addition of enzyme conjugates and substrate solutions according to the respective assay instructions. The enzymatic reaction was terminated with stop solution, and optical density was recorded at 450 nm using a microplate reader. Concentrations of each analyte were determined from the corresponding calibration curves and reported in the units specified by the manufacturers.

Total antioxidant status (TAS) and total oxidant status (TOS) were measured using commercial assay kits (Rel Assay Diagnostics, Gaziantep, Türkiye) on a Beckman Coulter AU5800 automated biochemical analyzer (Beckman Coulter, Inc., Brea, CA, USA) following the procedures described by Erel [56,57]. AS results are expressed as mmol Trolox equivalent/L, whereas TOS values are reported as μmol H_2_O_2_ equivalent/L. The oxidative stress index (OSI) was subsequently calculated as the ratio of TOS to TAS according to the following formula: OSI = (TOS (μmol H_2_O_2_ Eq/L)/TAS (mmol Trolox Eq/L)) × 100. The calculated OSI values are expressed as arbitrary units (AU) [58].

### 4.8. Histopathological Examination

Following macroscopic evaluation, distal colon tissues were fixed in 10% neutral buffered formalin, washed under running tap water for 12 h, and processed routinely for paraffin embedding. Tissue sections were prepared and stained with hematoxylin and eosin (H&E) for histopathological evaluation. All sections were examined by a blinded histopathologist who was unaware of the experimental groups. Histological alterations, including inflammatory cell infiltration, mucosal damage, ulcer formation, and crypt architectural distortion, were evaluated using a semiquantitative scoring system to determine the severity of experimental colitis [59].

### 4.9. Immunohistochemical Analysis of TGF-β and FGF Expression

Immunohistochemical analyses were performed on 4-μm-thick paraffin-embedded sections of colon tissue. After routine deparaffinization and rehydration, heat-induced antigen retrieval was carried out in citrate buffer (pH 6.0) using a microwave oven. Endogenous peroxidase activity was subsequently quenched with 3% hydrogen peroxide. The tissue sections were incubated with primary antibodies against TGF-β (Cat. No. sc-130348, Santa Cruz Biotechnology, Dallas, TX, USA) and FGF (Cat. No. sc-365106, Santa Cruz Biotechnology, Dallas, TX, USA). Following primary antibody incubation, a biotinylated secondary antibody (Cat. No. TP-015-HA, Thermo Fisher Scientific, Waltham, MA, USA) was applied, and immunoreactivity was detected using a streptavidin–peroxidase system (Cat. No. TP-015-HA, Thermo Fisher Scientific, Waltham, MA, USA). Color development was achieved with 3,3’-diaminobenzidine (DAB) (Cat. No. 34002, Thermo Fisher Scientific, Waltham, MA, USA), after which the sections were counterstained with hematoxylin. All stained slides were examined independently by a histopathologist who was blinded to the experimental groups. Immunoreactivity was evaluated using a semiquantitative scoring method based on staining intensity [60].

### 4.10. Quantitative Immunohistochemical Analysis

Quantitative analysis of TGF-β and FGF immunoreactivity was performed using QuPath software (version 0.5.1, University of Edinburgh, UK) under standardized analytical conditions. Whole-slide digital images were imported into the software, and representative regions of interest (ROIs) encompassing the colonic mucosal epithelium and lamina propria were manually selected while excluding tissue folds, necrotic regions, and processing artifacts. For each specimen, at least five randomly selected non-overlapping high-power fields (×400) were analyzed. Positive DAB staining was identified using the built-in color deconvolution algorithm with identical threshold settings applied to all sections. Staining intensity was automatically classified as negative (0), weak (1+), moderate (2+), or strong (3+). An H-score was calculated according to the following formula: H-score = (1 × % weakly stained cells) + (2 × % moderately stained cells) + (3 × % strongly stained cells), yielding a total score ranging from 0 to 300.

In addition, the percentage of DAB-positive stained area was determined for each section. Quantitative analyses were independently performed by two blinded observers, and the mean values were used for subsequent statistical analyses [61].

### 4.11. Protein–Protein Interaction Network Analysis

To explore the molecular interactions underlying inflammation and tissue repair in UC, a protein–protein interaction (PPI) network was established using the STRING database (Search Tool for the Retrieval of Interacting Genes/Proteins, version 12.0; https://string-db.org). A panel of 20 proteins (TNF, IL1B, IL6, TGFB1, FGF2, NFKB1, RELA, MAPK1, MAPK3, STAT3, AKT1, JUN, MMP9, VEGFA, EGFR, SMAD2, SMAD3, SMAD7, FGFR1, and FGFR2) was selected because of their documented involvement in inflammatory signaling, oxidative stress, and mucosal healing. The interaction network was generated for Rattus norvegicus using a minimum confidence score of 0.400. The resulting PPI dataset was subsequently exported to Cytoscape (version 3.10.4), where both network visualization and topological analyses were performed. Network architecture was characterized by calculating the number of nodes and edges, average node degree, clustering coefficient, network density, centralization, heterogeneity, and characteristic path length using the integrated NetworkAnalyzer plugin (version 4.4.8). To identify the most influential proteins within the network, hub genes were determined according to degree centrality.

This bioinformatic analysis was performed to complement the experimental findings by identifying key molecular interactions and signaling pathways that may contribute to the protective effects of quince gel in experimental UC [62,63].

### 4.12. Molecular Docking Analysis

Following LC–MS/MS profiling, quercetin was chosen as the representative phytochemical of quince gel for the molecular docking analysis. Its binding potential was investigated against proteins involved in inflammatory signaling and mucosal repair, including TNF-α, IL-1β, IL-6, TGF-β1, and FGF2.

Three-dimensional structures of the target proteins were obtained from the Protein Data Bank (PDB), whereas the chemical structure of quercetin was downloaded from the PubChem database. Prior to docking, both receptor and ligand structures were prepared by removing crystallographic water molecules, adding hydrogen atoms, and assigning Gasteiger charges.

Docking simulations were conducted using AutoDock Vina (version 1.2.5), and the binding conformation exhibiting the most favorable (lowest) binding free energy was selected as the optimal pose. The resulting protein–ligand complexes were then examined to characterize the binding mode and identify the amino acid residues participating in ligand recognition, thereby providing structural insight into the potential molecular mechanisms underlying the biological activity of quercetin [64,65].

### 4.13. Statistical Analysis

Statistical analyses were performed using IBM SPSS Statistics version 27.0 (IBM Corp., Armonk, NY, USA). Data distribution was assessed using the Shapiro–Wilk test together with visual inspection of histograms and Q–Q plots. Variables with a normal distribution are presented as mean ± standard deviation (SD) and were analyzed using one-way analysis of variance (ANOVA) followed by Tukey’s post hoc test. Variables that did not meet the normality assumption are presented as median (interquartile range, IQR) and were analyzed using the Kruskal–Wallis test followed by pairwise Mann–Whitney U tests with Bonferroni correction for multiple comparisons. A two-sided *p* value < 0.05 was considered statistically significant unless otherwise specified. Sample size estimation was performed prior to the study using G*Power software (version 3.1). Based on an anticipated effect size of 0.70 according to Cohen’s criteria, a significance level (α) of 0.05, and a statistical power of 80%, the minimum required sample size was calculated as 28 animals, corresponding to seven rats per experimental group.

## 5. Conclusions

In conclusion, the present study demonstrated that intrarectal administration of quince gel effectively alleviated experimental UC by reducing oxidative stress, attenuating inflammatory cytokine production, preserving colonic histological architecture, and modulating TGF-β- and FGF-associated mucosal repair responses. The identification of quercetin as one of the major phytochemical constituents, together with the complementary findings from protein–protein interaction network analysis and molecular docking, provides computational support for potential molecular mechanisms underlying the observed biological effects. Collectively, these experimental and in silico findings suggest that quince gel may represent a promising natural therapeutic candidate for the management of UC. However, the proposed molecular mechanisms require further experimental validation, and additional preclinical and well-designed clinical studies are needed to confirm its therapeutic potential and facilitate its translation into clinical practice.

## Figures and Tables

**Table 1:** Major phytochemical constituents identified in quince gel by LC–MS/MS.

Peak No.	Compound	RT (min)	Molecular Ion (*m*/*z*)	Relative Peak Intensity
1	Caffeic acid	4.81	179.03	Moderate
2	Chlorogenic acid	5.62	353.08	Moderate–high
3	Rutin	7.95	609.14	High
4	Quercetin	8.74	301.03	Very high (major peak)
5	Kaempferol	9.26	285.04	High

Relative peak intensity was visually estimated from the total ion chromatogram (TIC) and is presented to indicate the relative abundance of the detected phytochemicals rather than absolute quantitative concentrations.

**Table 2:** Oxidative stress parameters in the experimental groups.

Parameter	Sham	Quince Gel	UC	UC + Quince Gel	*p*
TAS (mmol Trolox Eq/L)	0.71 (0.68–0.81) ^a^	0.79 (0.74–0.82) ^a^	0.58 (0.55–0.61) ^c^	0.63 (0.61–0.65) ^b^	<0.001
TOS (μmol/L)	23.43 (5.76–48.49) ^ab^	11.00 (5.03–14.35) ^a^	79.65 (63.06–217.56) ^c^	41.77 (20.74–52.96) ^b^	0.001
OSI	3209.6 (847.1–7347.0) ^ab^	1392.4 (697.7–1857.4) ^a^	13,585.2 (11,016.5–35,665.6) ^c^	6847.5 (3395.4–9173.7) ^b^	<0.001
MDA (nmol/mL)	5.62 (5.35–6.02) ^ab^	5.16 (5.08–5.26) ^a^	7.57 (6.63–10.12) ^c^	6.22 (6.11–6.53) ^b^	<0.001

Data are presented as median (interquartile range). Overall group comparisons were performed using the Kruskal–Wallis test. Pairwise comparisons were performed using Mann–Whitney U tests with Bonferroni correction (adjusted significance threshold *p* < 0.0083). Groups sharing at least one common superscript letter are not significantly different, whereas groups with different superscript letters differ significantly.

**Table 3:** Biochemical and inflammatory parameters in the experimental groups.

Parameter	Sham	Quince Ge.	UC	UC + Quince Gel	*p*
Calcium (mg/dL)	11.30 (10.90–11.60) ^a^	11.10 (11.00–11.70) ^a^	26.50 (24.60–30.40) ^c^	16.20 (15.50–18.00) ^b^	<0.001
Albumin (g/L)	36.75 (33.88–42.48) ^a^	38.17 (35.30–40.62) ^a^	29.63 (27.88–31.84) ^b^	33.62 (32.45–34.67) ^ab^	0.002
CRP (mg/L)	0.11 (0.05–0.15) ^a^	0.06 (0.05–0.19) ^a^	0.34 (0.27–0.39) ^c^	0.19 (0.16–0.22) ^b^	0.001
LDH (U/L)	1248 (1020–1450) ^a^	1519 (1352–1608) ^ab^	2869.5 (2142–3348) ^c^	1678 (1562–1845) ^b^	0.001

Data are presented as median (interquartile range). Overall group comparisons were performed using the Kruskal–Wallis test. Pairwise comparisons were performed using Mann–Whitney U tests with Bonferroni correction (adjusted significance threshold *p* < 0.0083). Groups sharing at least one common superscript letter are not significantly different, whereas groups with different superscript letters differ significantly.

**Table 4:** Macroscopic, histopathological, and inflammatory parameters of the experimental groups.

Parameter	Sham	Quince Gel	UC	UC + Quince Gel	*p*
Colon Mass Index	0.55 (0.49–0.58) ^ab^	0.48 (0.41–0.51) ^a^	0.625 (0.61–0.64) ^c^	0.57 (0.55–0.58) ^bc^	0.001
Serum TNF-α	9 (8–13) ^a^	7 (6–10) ^a^	83 (67–96) ^c^	56 (43–60) ^b^	<0.001
Serum IL-1β	8 (7–8) ^a^	7 (5–9) ^a^	90 (71–101) ^c^	64 (55–69) ^b^	<0.001
Serum IL-6	19 (15–25) ^a^	12 (11–16) ^a^	128.5 (99–154) ^c^	58 (49–64) ^b^	<0.001
Macroscopic damage score	0 (0–1) ^a^	0 (0–0) ^a^	3 (3–3) ^c^	1 (1–2) ^b^	<0.001
Histopathological score	0 (0–1) ^a^	1 (0–1) ^a^	2 (2–3) ^c^	1 (1–2) ^ab^	0.001

Data are presented as median (interquartile range). Overall group comparisons were performed using the Kruskal–Wallis test. Pairwise comparisons were performed using Mann–Whitney U tests with Bonferroni correction (adjusted significance threshold *p* < 0.0083). Groups sharing at least one common superscript letter are not significantly different, whereas groups with different superscript letters differ significantly.

**Table 5:** Quantitative immunohistochemical analysis of TGF-β and FGF expression.

Parameter	Sham	Quince Gel	UC	UC + Quince Gel	*p*
TGF-β-Positive Area (%)	7.4 ± 1.8 ^a^	8.2 ± 2.0 ^a^	36.7 ± 5.4 ^c^	20.5 ± 3.6 ^b^	<0.001
TGF-β H-score	48.3 ± 9.5 ^a^	52.1 ± 10.2 ^a^	175.8 ± 18.9 ^c^	101.4 ± 14.7 ^b^	<0.001
FGF-Positive Area (%)	6.9 ± 1.5 ^a^	7.3 ± 1.7 ^a^	33.5 ± 4.9 ^c^	18.7 ± 3.2 ^b^	<0.001
FGF H-score	44.2 ± 8.7 ^a^	46.8 ± 9.1 ^a^	168.2 ± 17.4 ^c^	96.3 ± 13.2 ^b^	<0.001

Data are presented as mean ± SD. Overall group comparisons were performed using one-way ANOVA followed by Tukey’s multiple comparison test. Groups sharing at least one common superscript letter are not significantly different, whereas groups with different superscript letters differ significantly (*p* < 0.05).

**Table 6:** Top ten hub genes identified by degree centrality analysis in the protein–protein interaction network.

Rank	Gene	Degree
1	TGFB1	19
2	MMP9	19
3	STAT3	19
4	IL6	18
5	MAPK3	18
6	AKT1	18
7	IL1B	17
8	JUN	16
9	EGFR	16
10	FGF2	16

The prominent connectivity of inflammation-related cytokines and growth factor-associated proteins within the network suggests close functional interactions among signaling pathways involved in inflammatory responses and mucosal repair in UC.

**Table 7:** Predicted binding affinities of quercetin against inflammation- and tissue remodeling-related target proteins obtained by molecular docking analysis.

Ligand	Target Protein	Binding Affinity (kcal/mol)
Quercetin	TNF-α	−7.379
Quercetin	TGF-β1	−7.059
Quercetin	IL1 β	−6.913
Quercetin	IL-6	−6.434
Quercetin	FGF2	−5.682

## Data Availability

The original contributions presented in this study are included in the article/Supplementary Materials. Further inquiries can be directed to the corresponding author.

## Supplementary Materials

The following supporting information can be downloaded at: https://www.mdpi.com/article/10.3390/ph19081161/s1, Figure S1: Representative total ion chromatogram (TIC) of quince gel obtained by LC–MS/MS (ESI−).

## References

[1] Si X.Y., Merlin D., Xiao B. (2016). Recent advances in orally administered cell-specific nanotherapeutics for inflammatory bowel disease. World J. Gastroenterol..

[2] Liang Y., Li Y., Lee C., Yu Z., Chen C., Liang C. (2024). Ulcerative colitis: Molecular insights and intervention therapy. Mol. Biomed..

[3] Jia J., Liu Y., Wang D., Pan Z., Zheng Q., Lu J., Liang C., Li D. (2026). Ulcerative colitis: Signaling pathways, therapeutic targets and interventional strategies. Signal Transduct. Target. Ther..

[4] Cha H., Lee S., Hwan Kim S., Kim H., Lee D.S., Lee H.S., Lee J.H., Park J.W. (2017). Increased susceptibility of IDH2-deficient mice to dextran sodium sulfate-induced colitis. Redox Biol..

[5] Kumar V.L., Pandey A., Verma S., Das P. (2019). Protection afforded by methanol extract of Calotropis procera latex in experimental model of colitis is mediated through inhibition of oxidative stress and pro-inflammatory signaling. Biomed. Pharmacother..

[6] Bu F., Chen K., Chen S., Jiang Y. (2025). Gut microbiota and intestinal immunity interaction in ulcerative colitis and its application in treatment. Front. Cell. Infect. Microbiol..

[7] Muro P., Zhang L., Li S., Zhao Z., Jin T., Mao F., Mao Z. (2024). The emerging role of oxidative stress in inflammatory bowel disease. Front. Endocrinol..

[8] Manful C.F., Fordjour E., Subramaniam D., Sey A.A., Abbey L., Thomas R. (2025). Antioxidants and Reactive Oxygen Species: Shaping Human Health and Disease Outcomes. Int. J. Mol. Sci..

[9] Jones M.K., Tomikawa M., Mohajer B., Tarnawski A.S. (1999). Gastrointestinal mucosal regeneration: Role of growth factors. Front. Biosci..

[10] Son B. (2025). Regulation of Tissue Regeneration by Immune Microenvironment-Fibroblast Interactions. Int. J. Mol. Sci..

[11] Stolfi C., Troncone E., Marafini I., Monteleone G. (2020). Role of TGF-Beta and Smad7 in Gut Inflammation, Fibrosis and Cancer. Biomolecules.

[12] Frangogiannis N. (2020). Transforming growth factor-β in tissue fibrosis. J. Exp. Med..

[13] Lian N., Li T. (2016). Growth factor pathways in hypertrophic scars: Molecular pathogenesis and therapeutic implications. Biomed. Pharmacother..

[14] Tahir U.F., Noureen S., Partab F., Afzal M.W., Afzal M., Mehmood A., Afzal F., Hasan K., Sameeha F., Riaz S. (2026). A Comparison of Biological Therapies vs Traditional Immunosuppressant in the Management of Inflammatory Bowel Diseases: A Narrative Review. J. Community Hosp. Intern. Med. Perspect..

[15] Barbosa Bezerra G., de Menezes de Souza L., Dos Santos A.S., de Almeida G.K., Souza M.T., Santos S.L., Aparecido Camargo E., Dos Santos Lima B., de Souza Araujo A.A., Cardoso J.C. (2017). Hydroalcoholic extract of Brazilian red propolis exerts protective effects on acetic acid-induced ulcerative colitis in a rodent model. Biomed. Pharmacother..

[16] Ashraf M.U., Muhammad G., Hussain M.A., Bukhari S.N. (2016). *Cydonia oblonga M*., A Medicinal Plant Rich in Phytonutrients for Pharmaceuticals. Front. Pharmacol..

[17] Najman K., Adrian S., Sadowska A., Swiader K., Hallmann E., Buczak K., Waszkiewicz-Robak B., Szterk A. (2023). Changes in Physicochemical and Bioactive Properties of Quince (*Cydonia oblonga* Mill.) and Its Products. Molecules.

[18] Tamri P., Hemmati A., Boroujerdnia M.G. (2014). Wound healing properties of quince seed mucilage: In vivo evaluation in rabbit full-thickness wound model. Int. J. Surg..

[19] Ermiş I.S., Deveci E., Aşır F. (2023). Effects of Quince Gel and Hesperidin Mixture on Experimental Endometriosis. Molecules.

[20] Minaiyan M., Ghannadi A., Etemad M., Mahzouni P. (2012). A study of the effects of *Cydonia oblonga* Miller (Quince) on TNBS-induced ulcerative colitis in rats. Res. Pharm. Sci..

[21] Parvan M., Sajjadi S.E., Minaiyan M. (2017). Protective Effect of Two Extracts of *Cydonia oblonga* Miller (Quince) Fruits on Gastric Ulcer Induced by Indomethacin in Rats. Int. J. Prev. Med..

[22] Essafi-Benkhadir K., Refai A., Riahi I., Fattouch S., Karoui H., Essafi M. (2012). Quince (*Cydonia oblonga* Miller) peel polyphenols modulate LPS-induced inflammation in human THP-1-derived macrophages through NF-κB, p38MAPK and Akt inhibition. Biochem. Biophys. Res. Commun..

[23] Herrera-Rocha K.M., Rocha-Guzmán N.E., Gallegos-Infante J.A., González-Laredo R.F., Larrosa-Pérez M., Moreno-Jiménez M.R. (2022). Phenolic Acids and Flavonoids in Acetonic Extract from Quince (*Cydonia oblonga* Mill.): Nutraceuticals with Antioxidant and Anti-Inflammatory Potential. Molecules.

[24] Zhang P., Jiao H., Wang C., Lin Y., You S. (2019). Chlorogenic Acid Ameliorates Colitis and Alters Colonic Microbiota in a Mouse Model of Dextran Sulfate Sodium-Induced Colitis. Front. Physiol..

[25] Wan F., Zhong R., Wang M., Zhou Y., Chen Y., Yi B., Hou F., Liu L., Zhao Y., Chen L. (2021). Caffeic Acid Supplement Alleviates Colonic Inflammation and Oxidative Stress Potentially Through Improved Gut Microbiota Community in Mice. Front. Microbiol..

[26] Wang Z., Li S., Cao Y., Tian X., Zeng R., Liao D.F., Cao D. (2016). Oxidative Stress and Carbonyl Lesions in Ulcerative Colitis and Associated Colorectal Cancer. Oxid. Med. Cell. Longev..

[27] Quattrini S., Galeazzi T., Monachesi C., Palpacelli A., Catassi G., Quatraccioni C., Annulli G., Di Sario A., Cianfruglia L., Orciani M. (2026). The Oxidative Stress Imbalance in Children and Adults with IBD and Associated Factors. Nutrients.

[28] Yi J.-L., Zhu J.-X., Huang W.-F., Yi L.-T. (2026). Mechanistic Networks, Cellular Specificity, and Therapeutic Opportunities of Ferroptosis in Ulcerative Colitis. Pharmaceuticals.

[29] Tan C., Fan H., Ding J., Han C., Guan Y., Zhu F., Wu H., Liu Y., Zhang W., Hou X. (2022). ROS-responsive nanoparticles for oral delivery of luteolin and targeted therapy of ulcerative colitis by regulating pathological microenvironment. Mater. Today Bio.

[30] Rana S.V., Sharma S., Prasad K.K., Sinha S.K., Singh K. (2014). Role of oxidative stress & antioxidant defence in ulcerative colitis patients from north India. Indian J. Med. Res..

[31] Sahoo D.K., Heilmann R.M., Paital B., Patel A., Yadav V.K., Wong D., Jergens A.E. (2023). Oxidative stress, hormones, and effects of natural antioxidants on intestinal inflammation in inflammatory bowel disease. Front. Endocrinol..

[32] Gubitosa F., Fraternale D., De Bellis R., Gorassini A., Benayada L., Chiarantini L., Albertini M.C., Potenza L. (2023). *Cydonia oblonga* Mill. Pulp Callus Inhibits Oxidative Stress and Inflammation in Injured Cells. Antioxidants.

[33] Boulebd H., Carmena-Bargueño M., Pérez-Sánchez H. (2023). Exploring the Antioxidant Properties of Caffeoylquinic and Feruloylquinic Acids: A Computational Study on Hydroperoxyl Radical Scavenging and Xanthine Oxidase Inhibition. Antioxidants.

[34] Yang D., Wang T., Long M., Li P. (2020). Quercetin: Its Main Pharmacological Activity and Potential Application in Clinical Medicine. Oxid. Med. Cell. Longev..

[35] Tang X., Huang Y., Zhu Y., Xu Y. (2025). Immune dysregulation in ulcerative colitis: Pathogenic mechanisms and therapeutic strategies of traditional Chinese medicine. Front. Cell Dev. Biol..

[36] Kurimoto M., Watanabe T., Kamata K., Minaga K., Kudo M. (2021). IL-33 as a Critical Cytokine for Inflammation and Fibrosis in Inflammatory Bowel Diseases and Pancreatitis. Front. Physiol..

[37] Neurath M.F. (2024). Strategies for targeting cytokines in inflammatory bowel disease. Nat. Rev. Immunol..

[38] Strober W., Fuss I.J. (2011). Proinflammatory cytokines in the pathogenesis of inflammatory bowel diseases. Gastroenterology.

[39] Chang L., Zhang X.X., Ren Y.P., Cao L., Zhi X.R., Zhang L.T. (2013). Simultaneous Quantification of Six Major Flavonoids From Fructus sophorae by LC-ESI-MS/MS and Statistical Analysis. Indian J. Pharm. Sci..

[40] Prudovsky I. (2021). Cellular Mechanisms of FGF-Stimulated Tissue Repair. Cells.

[41] Sommer K., Wiendl M., Müller T.M., Heidbreder K., Voskens C., Neurath M.F., Zundler S. (2021). Intestinal Mucosal Wound Healing and Barrier Integrity in IBD-Crosstalk and Trafficking of Cellular Players. Front. Med..

[42] Beck P.L., Rosenberg I.M., Xavier R.J., Koh T., Wong J.F., Podolsky D.K. (2003). Transforming growth factor-beta mediates intestinal healing and susceptibility to injury in vitro and in vivo through epithelial cells. Am. J. Pathol..

[43] Rodari M.M., Cerf-Bensussan N., Parlato M. (2022). Dysregulation of the immune response in TGF-β signalopathies. Front. Immunol..

[44] Liu X., Jing M., Yang Y., Jin Q., Feng B., Zhang P., Niu C., Hu X., Huang Z. (2026). FGF family in health and disease. Mol. Biomed..

[45] Borowczak J., Szczerbowski K., Maniewski M., Kowalewski A., Janiczek-Polewska M., Szylberg A., Marszałek A., Szylberg Ł. (2022). The Role of Inflammatory Cytokines in the Pathogenesis of Colorectal Carcinoma—Recent Findings and Review. Biomedicines.

[46] Souza R.F., Caetano M.A.F., Magalhães H.I.R., Castelucci P. (2023). Study of tumor necrosis factor receptor in the inflammatory bowel disease. World J. Gastroenterol..

[47] National Research Council (2011). Guide for the Care and Use of Laboratory Animals: Eighth Edition.

[48] Tuba, Hussain M.A., Muhammad G., Raza M.A., Ashraf A., Haseeb M.T., Mushtaq M., Shafiq Z. (2025). A comprehensive review on phytochemistry, pharmacology, preclinical, and clinical trials of *Cydonia oblonga*. Phytochem. Rev..

[49] Adamovic A., Tomovic M., Andjic M., Dimitrijevic J., Glisic M., Adamovic M. (2025). *Cydonia oblonga*: A Comprehensive Overview of Applications in Dermatology and Cosmetics. Cosmetics.

[50] Hassanshahi N., Masoumi S.J., Mehrabani D., Hashemi S.S., Zare M. (2020). The Healing Effect of Aloe Vera Gel on Acetic Acid-Induced Ulcerative Colitis in Rat. Middle East J. Dig. Dis..

[51] Mohamed N.I., El-Kashef D.H., Suddek G.M. (2022). Flavocoxid halts both intestinal and extraintestinal alterations in acetic acid-induced colitis in rats. Environ. Sci. Pollut. Res. Int..

[52] Baydi Z., Limami Y., Khalki L., Zaid N., Naya A., Mtairag E.M., Oudghiri M., Zaid Y. (2021). An Update of Research Animal Models of Inflammatory Bowel Disease. Sci. World J..

[53] Randhawa P.K., Singh K., Singh N., Jaggi A.S. (2014). A review on chemical-induced inflammatory bowel disease models in rodents. Korean J. Physiol. Pharmacol..

[54] Soliman S.M., Wadie W., Shouman S.A., Ainshoka A.A. (2018). Sodium selenite ameliorates both intestinal and extra-intestinal changes in acetic acid-induced colitis in rats. Naunyn Schmiedebergs Arch. Pharmacol..

[55] Nazari-Khanamiri F., Jafari A., Esmaeilzadeh Z., Ghasemnejad-Berenji M. (2023). Biochemical and histopathological evidence for beneficial effects of Empagliflozin pretreatment on acetic acid-induced colitis in rats. BMC Gastroenterol..

[56] Erel O. (2005). A new automated colorimetric method for measuring total oxidant status. Clin. Biochem..

[57] Erel O. (2004). A novel automated direct measurement method for total antioxidant capacity using a new generation, more stable ABTS radical cation. Clin. Biochem..

[58] Motor S., Ozturk S., Ozcan O., Gurpinar A.B., Can Y., Yuksel R., Yenin J.Z., Seraslan G., Ozturk O.H. (2014). Evaluation of total antioxidant status, total oxidant status and oxidative stress index in patients with alopecia areata. Int. J. Clin. Exp. Med..

[59] Aşır F., Gökalp Özkorkmaz E., Yalçın M., Şahin F., Korak T. (2026). Skimmianine Pretreatment Attenuates Cerebellar Neuroinflammation and Myelin Injury Following Experimental Cerebral Ischemia–Reperfusion. Antioxidants.

[60] Guler R., Ozden E., Asır F., Gulsun B. (2025). High-Dose Shilajit Enhances Xenograft-Mediated Bone Regeneration in a Rat Tibial Defect Model: An In Vivo Experimental Study. Life.

[61] Aşır F., Erdemci F., Çankırı Z., Korak T., Başaran S.Ö., Kaplan Ö., Yükselmiş Ö., Dönmezdil N., Ayaz H., Kaplan Ş. (2024). Zonisamide Ameliorated the Apoptosis and Inflammation in Cerebellar Tissue of Induced Alcohol Addiction Animal Model. Life.

[62] Kaplan Ö., Karabat M.U., Özdemir Başaran S., Yavuz D., Aşır F., Korak T., Ağaçayak E., Deveci E. (2026). Altered Endocannabinoid Signaling in Placentas from SARS-CoV-2-Infected Pregnancies. Diagnostics.

[63] Keşim D.A., Aşır F., Ayaz H., Korak T. (2024). The Effects of Ellagic Acid on Experimental Corrosive Esophageal Burn Injury. Curr. Issues Mol. Biol..

[64] Karaaslanlı A., Tuncer M.C., Aşır F., Korak T. (2025). Gallic acid showed neuroprotection against endoplasmic reticulum stress in rats. Acta Cir. Bras..

[65] Korak T., Ayaz H., Aşır F. (2025). Skimmianine Modulates Tumor Proliferation and Immune Dynamics in Breast Cancer by Targeting PCNA and TNF-α. Pharmaceuticals.

