# Supplementary material for: Quince Gel Attenuates Experimental Ulcerative Colitis Through Antioxidant, Anti-Inflammatory and Mucosal Repair Mechanisms: Integrated Histopathological, Bioinformatic and Molecular Docking Analyses
Source: Pharmaceuticals (Basel). 2026 Jul 25;19(8):1161. doi: 10.3390/ph19081161 (PMC13516655; doi:10.3390/ph19081161)
Supplement: Supplementary file 1 [file pharmaceuticals-19-01161-s001.zip › pharmaceuticals-4405328-supplementary.pdf]

Supplementary Figures

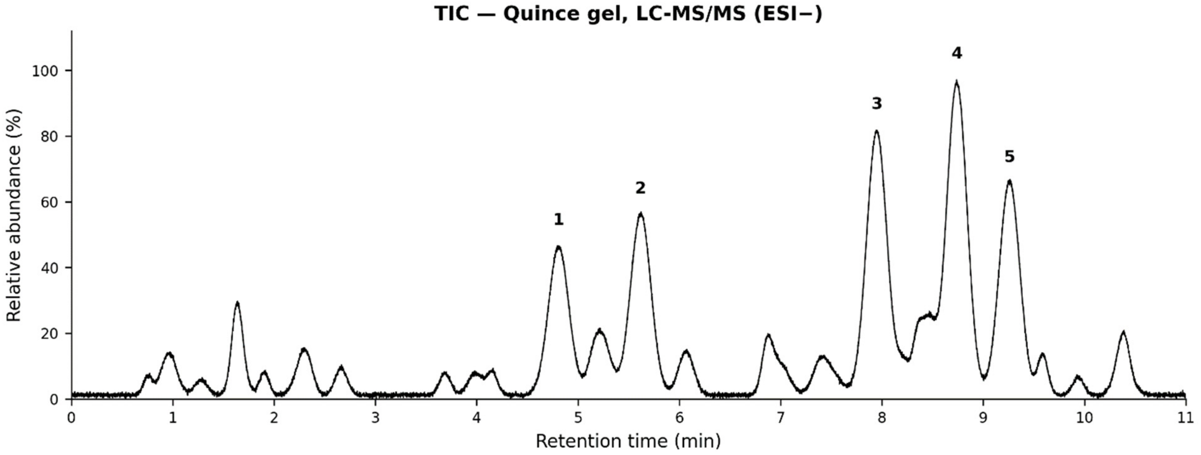

Figure S1. Representative total ion chromatogram (TIC) of quince gel obtained by LC–MS/MS (ESI−). Only the major identified phytochemical peaks are labeled: (1) caffeic acid, (2) chlorogenic acid, (3) rutin, (4) quercetin, and (5) kaempferol. Minor peaks correspond to additional low-abundance constituents that were not further characterized in the present study.

1. Caffeic acid RT 4.81 min, m/z 179.032. Chlorogenic acid RT 5.62 min, m/z 353.08 3. Rutin RT 7.95 min, m/z 609.144. Quercetin RT 8.74 min, m/z 301.035. Kaempferol RT 9.26 min, m/z 285.04
